# Development of a rat capnoperitoneum phantom to study drug aerosol deposition in the context of anticancer research on peritoneal carcinomatosis

**DOI:** 10.1038/s41598-021-01332-0

**Published:** 2021-11-08

**Authors:** Daniel Göhler, Antje Geldner, Ralf Gritzki, Franz Lohse, Stephan Große, Julien Sobilo, Clemens Felsmann, Jonathan R. Buggisch, Alain Le Pape, Andreas Rudolph, Michael Stintz, Urs Giger-Pabst

**Affiliations:** 1 Research Group Mechanical Process Engineering, Institute of Process Engineering and Environmental Technology, Technische Universität Dresden, 01062 Dresden, Germany; 2 Topas GmbH, 01237 Dresden, Germany; 3 Process Systems Engineering Group, Institute of Process Engineering and Environmental Technology, Technische Universität Dresden, 01062 Dresden, Germany; 4 Chair of Building Energy Systems and Heat Supply, Institute of Power Engineering, Technische Universität Dresden, 01062 Dresden, Germany; 5 CNRS, TAAM UPS44, In Vivo Imaging Centre for Preclinical and Translational Research CIPA, 45071 Orléans, France; 6 Department of General-, Visceral- and Transplant Surgery, Universität Münster, Münster, Germany; 7 Surgical Clinic I, Mathias-Spital Rheine, 48431 Rheine, Germany

**Keywords:** Experimental models of disease, Biomedical engineering, Fluid dynamics, Experimental particle physics, Characterization and analytical techniques, Cancer therapy, Drug development

## Abstract

Pressurized Intraperitoneal Aerosol Chemotherapy (PIPAC) is a promising approach with a high optimization potential for the treatment of peritoneal carcinomatosis. To study the efficacy of PIPAC and drugs, first rodent cancer models were developed. But inefficient drug aerosol supply and knowledge gaps concerning spatial drug distribution can limit the results based on such models. To study drug aerosol supply/deposition, computed tomography scans of a rat capnoperitoneum were used to deduce a virtual and a physical phantom of the rat capnoperitoneum (RCP). RCP qualification was performed for a specific PIPAC method, where the capnoperitoneum is continuously purged by the drug aerosol. In this context, also in-silico analyses by computational fluid dynamic modelling were conducted on the virtual RCP. The physical RCP was used for ex-vivo granulometric analyses concerning drug deposition. Results of RCP qualification show that aerosol deposition in a continuous purged rat capnoperitoneum depends strongly on the position of the inlet and outlet port. Moreover, it could be shown that the droplet size and charge condition of the drug aerosol define the deposition efficiency. In summary, the developed virtual and physical RCP enables detailed in-silico and ex-vivo analyses on drug supply/deposition in rodents.

## Introduction

Pressurized Intraperitoneal Aerosol Chemotherapy (PIPAC) was clinically introduced as a palliative treatment in 2011 to manage patients suffering from end-stage peritoneal carcinomatosis^1^ that is (i) diagnosed yearly for over 1 million patients worldwide^2^ and (ii) still the ultimate cause of death in most cases. During PIPAC, the chemotherapeutic drug is nebulized into a stable capnoperitoneum as a therapeutic aerosol. In contrast to other chemotherapeutic treatment approaches (e.g. systemic chemotherapy, intraperitoneal liquid chemotherapy or hyperthermic intraperitoneal chemoperfusion), PIPAC benefits from (i) a good system tolerability, (ii) only very few side effects, (iii) higher drug penetration into the tumoral tissue and (iv) induces histological regression of peritoneal carcinomatosis that is resistant to conventional liquid intraperitoneal or intravenous chemotherapy^3^. Accordingly, PIPAC becomes more and more popular all over the world^4^.

But as typical for new treatment approaches and technologies, there is also a high optimization potential for PIPAC^5,6^. This applies especially for (i) the aerosol characteristics (e.g. size, concentration, temperature and velocity of droplets)^7,8^, (ii) the homogeneity of the spatial drug distribution^7–9^, (iii) the kind and dose of chemotherapeutic drugs^10^ and (iv) the whole treatment procedure (e.g. method of drug aerosolization, position of aerosol supply, frequency and duration of treatment). Due to the lack of fundamental research studies, PIPAC is still seen as a highly empiric treatment approach^10^. Moreover, the superiority of PIPAC over other treatment procedures for peritoneal carcinomatosis is not proved yet^10^.

The geometric dimensions of the conventional PIPAC nozzle (i.e., outer nozzle diameter of 9 mm) as used for drug aerosolization in humans and the high quantity of drug solution (several tenth up to hundreds millilitres) for its operation allow to a large extent solely studies on large animals like pigs or on the base of organic^11,12^ (i.e., inverted bovine urinary bladder) and simple inorganic phantoms^9^ (i.e., plastic boxes or barrels). To study the efficacy of PIPAC and drugs more detailed, two recently published studies^6,13^ used a rat peritoneal metastasis model based on human ovarian cancer cells (SKOV3)^14^. Both studies operated the conventional PIPAC technology to treat the animals, but the large wound to body size dimension has to be seen critical^13^. For the purpose of illustration, a scaling of the rat wound dimension by the ratio of the capnoperitoneal volume of humans to that of rats (volume ratio of approx. 17–20) would lead for humans to a considerably wound dimension of 20–24 cm. To prevent such large wound dimensions, more suitable approaches for performing PIPAC in rodents are required in parallel to the cancer models. Modified PIPAC approaches for rodents were designed for example in parallel to a mouse ovarian cancer model (OVCAR3 and SKOV3)^15^.

Mindless of conventional or modified PIPAC approaches, there are a lot of knowledge gaps concerning drug aerosol supply, propagation and deposition, which can be analysed without the use of animals or humans by means of adequate animal or human replacement models. Accordingly, we started to develop a static rat capnoperitoneum phantom (RCP) in virtual and physical form to study drug aerosol supply and deposition. The former one serves for in-silico computational fluid dynamic modelling similar to the use in other organs^16–18^, while the latter one was realized by 3D filament printing^19,20^ and used for ex-vivo/situ aerosol analysis.

## Methods

### Ethical approval and statement

In-vivo analyses were approved by the French Ministry of Higher Education, Research and Innovation (Ministère de l’Enseignement supérieur, de la Recherche et de l'Innovation, France) under registration #18904-201901301606953v1 and #21574-201901301606953v3. Animals were handled and cared in accordance with national and institutional guidelines. Protocols were conducted by authorized investigators. All methods are reported in accordance with ARRIVE guidelines^21^.

### Design of the virtual and the physical rat capnoperitoneum phantom (RCP)

**Figure 1:**
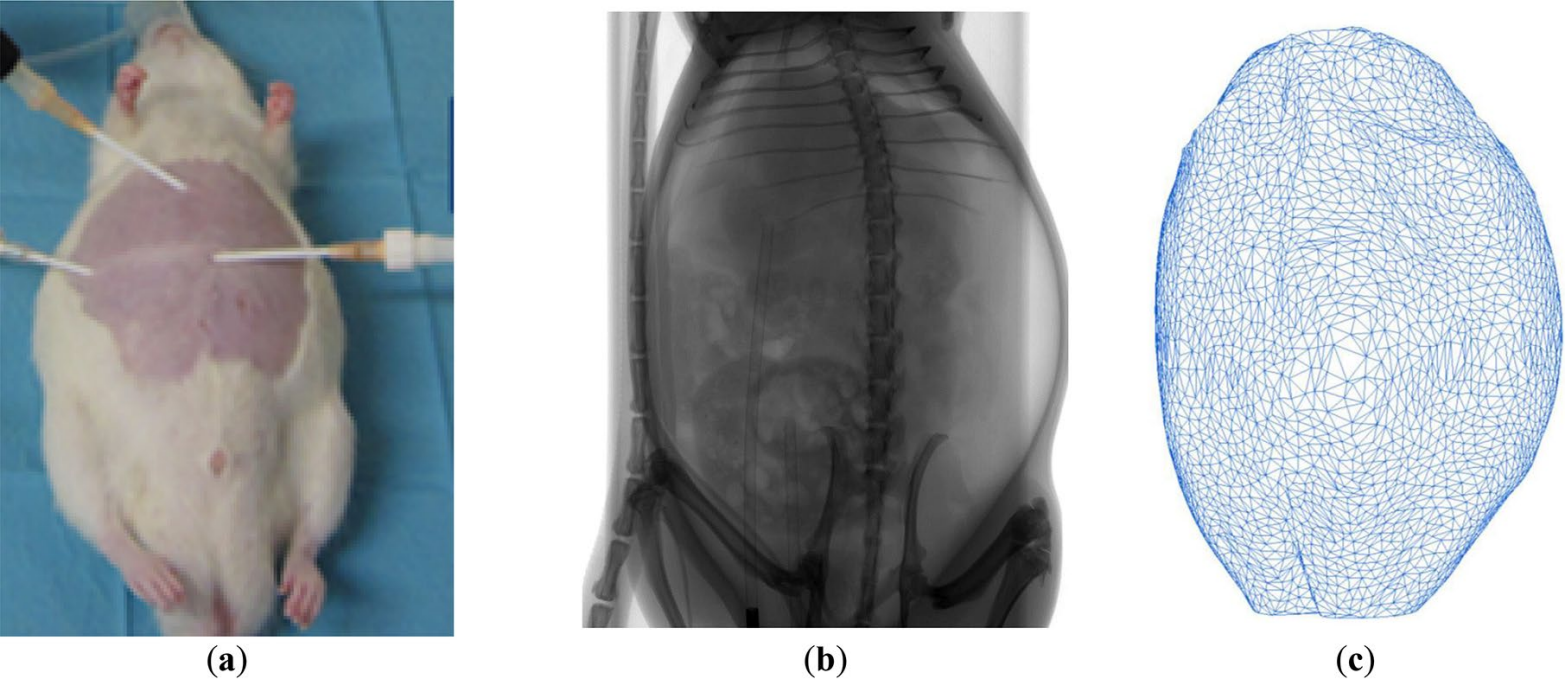
Phantom of the rat capnoperitoneum: (**a**) anesthetized rat, (**b**) respiration-triggered computed tomography scan of rat capnoperitoneum in supine position at a capnoperitoneal pressure of p_c,rat_ = 1.07 kPa = 8 mmHg; (**c**) surface mesh of final virtual rat capnoperitoneum phantom.

The basis for the development of the rat capnoperitoneum phantom (RCP) was a 275 g RNU rat (Crl: NIH-Foxn1rnu, Charles River, France) as used i.a. for anticancer research^6,14^. A representative rat capnoperitoneum (i.e., with carbon dioxide inflated abdominal cavity) based on a capnoperitoneal pressure of 8 mmHg = 1.07 kPa^6,13^ was established via a catheter (Surflow® I.V. Catheter 14G, ID SR + OX1464C1, Terumo) within a prior anesthetized rat (2% air/isoflurane mixture, Iso-Vet, Piramal Healthcare) as shown in Fig. 1a). Highly-resolved geometric data of the rat capnoperitoneum with an image resolution of 100 µm per pixel as shown in Fig. 1b) were recorded by respiration-triggered computed tomography (SkyScan 1278 micro CT system, Bruker Corporation, Billerica, USA). To improve the intelligibility, a CT video sequence (S1) of the rat capnoperitoneum is provided in the supplementary material.

Single, binarized CT image sequences were merged by means of scientific image processing software (ImageJ 1.52n, National Institutes of Health, USA)^22^ to reconstruct a virtual capnoperitoneum of the rat in form of a filled body object. Afterwards, a mesh processing program (MeshLab 2016)^23^ was used (i) to deduce a mesh of the surface structure of the rat capnoperitoneum and (ii) to revise the primary mesh (with more than 1.1 million surface elements) for further processing (e.g.: filling of wholes, removal of intersections, reduction of surface elements). The final virtual mesh (designated as virtual rat capnoperitoneum phantom) shows a capnoperitoneal volume of 146.2 mL and consists of 10′000 triangular surface elements as shown in Fig. 1c).

**Figure 2:**
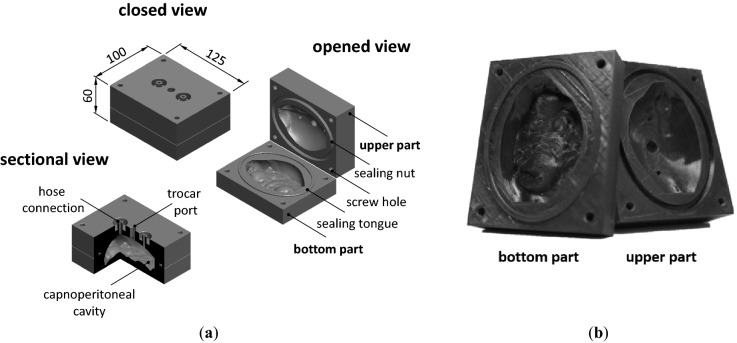
Rat capnoperitoneum phantom: (**a**) CAD model in different views and (**b**) photograph of bottom part (i.e., visceral peritoneum) and upper part (parietal peritoneum) of the model after additive manufacturing by 3D filament printing.

For the design of the physical RCP, the virtual RCP (in STL File format) was further processed by a commercial three-dimensional computer-aided design (CAD) software (SolidWorks 2017, Dassault Systèmes, Vélizy-Villacoublay, France). During that procedure, a cavity based on the virtual RCP surface structure was embedded in a full body cuboid (125 × 100 × 60) mm. Afterwards, the cuboid was (i) divided in two pieces (i.e., in a bottom part that contains mainly the visceral peritoneum with the irregular surface of the inner organs and an upper part that shows mainly the parietal peritoneum with the smooth abdominal wall) and (ii) equipped with 3 access ports for studying conventional PIPAC or modified PIPAC procedures. The final CAD model as shown in Fig. 2 was than printed over 15 h with a resolution of 400 µm by means of a filament 3D printer (modified Renkforce RF2000, Conrad Electronics, Hirschau, Germany) and a filament made of glycol modified polyethylene terephthalate. Afterwards, the inner surface of the physical model was smoothed with a sanding sponge (graining P220) prior assembly.

For the final physical RCP, a capnoperitoneal volume of (130.85 ± 3.89) mL was determined via volumetric analyses by water. The deviation of the capnoperitoneal volume of the physical RCP to the virtual RCP of − 10.5 vol.-% is attributed to (i) the tessellation of the surface mesh by converting the CAD model into the STL file format^24^ required for 3D printing, (ii) the coarse print size resolution of 400 µm, (iii) shrinking phenomena during printing and (iv) the calibration of the 3D printer.

### Studied scenarios for drug aerosol supply

In analogy to previous work^8^, a continuously purging of the phantoms by the “drug aerosol” was simulated. Different scenarios for drug aerosol supply were chosen to (i) characterise the performance of the virtual and the physical phantom and (ii) to study the impact of different measures (i.e., flow rates, purging direction, aerosol charge condition) on flow conditions and aerosol deposition. An overview on the investigated scenarios is given in Table 1.

**Table 1:** Studied scenarios for drug aerosol supply to the rat capnoperitoneum phantoms (RCP).

Phantom (–)	Volume (mL)	Flow rate (SL/min)	Pressure drop (mbar)	Residence time^a^ (s)	Purging direction (–)	Charge^b^ (–)
Virtual RCP	146.2 ± 0.00	1.0	n/a	8.77 ± 0.00	Top-down, bottom-up	n/a
		3.0		2.92 ± 0.00	Top-down, bottom-up	
		5.0		1.75 ± 0.00	Top-down, bottom-up	
Physical RCP	130.85 ± 3.89	1.0	0.08 ± 0.029	7.85 ± 0.24	Top-down	+/−, +
		3.0	0.60 ± 0.000	2.62 ± 0.08	Top-down	+/−, +
		5.0	1.72 ± 0.076	1.57 ± 0.05	Top-down	+/−, +
Alternative RCP	134.65 ± 0.43	1.0	0.07 ± 0.029	8.08 ± 0.03	Top-down	+
		3.0	0.43 ± 0.058	2.69 ± 0.01	Top-down	+
		5.0	0.88 ± 0.029	1.62 ± 0.01	Top-down	+

^a^Ratio of inner RCP volume and air/aerosol flow rate that describes the mean period of time a discrete volume passes the RCP.

^b^i.e., aerosol charge condition; + / − … bipolar neutralized aerosol, + … macroscopic positively charged aerosol.

According to Table 1, also an alternative RCP was operated for comparison purposes between simple and complex phantoms. The alternative RCP was just a laboratory glass bottle with a nominal volume of 100 mL (GL45 100 ml DURAN® glass bottle, DWK Life Science, Wertheim/Main, Germany), an outer diameter of approx. 56 mm, a height of 105 mm and a GL45 screw cap that contains two hose ports for aerosol supply and exhaust. Volumetric analyses by water reveal that the alternative phantom offers an inner volume of (134.65 ± 0.43) mL that does not deviate significantly from the inner volume of the physical RCP. Note that a conductive hose with a length of 45 mm at the inlet port within the alternative RCP served to mimic the distance between inlet and outlet port of the physical RCP.

### In-silico computational fluid dynamic (CFD) modelling

Fluid dynamic phenomena were computed for isothermal conditions by means of an in-house CFD research code (ParallelNS, Göttingen University and Technische Universität Dresden, Germany) that uses a Galerkin Least-Squares finite element approach to solve unsteady Reynolds-averaged Navier–Stokes equations^25^. Side considerations showed that the flow conditions in all scenarios are turbulent (i.e., Reynolds numbers at the inlet varied between 6100 and 30500). For the calculation of turbulence effects, two turbulence models were used, i.e., a k–ε model with specific wall functions (for momentum and turbulent transport quantities) and a φ–f–k–ε model without wall functions^26^. ParallelNS offers also a wide variety of additional transport equations like different mass transport equations or the equation of air age, respectively^27^.

The calculations for this study were performed in analogy to the experiments, i.e., with the same boundary conditions and experimental parameters as used during the ex-vivo aerosol analyses. Matching parameters of the turbulence variables were given (i.e., turbulence intensity of 10%, turbulent length scale of 0.001 m). For computation, the virtual RCP was endued with 1.03 million tetrahedron grid elements. Calculations were performed transient (time resolution of 0.1 s). In order to be able to reproduce more complicated test arrangements in future analyses, especially with regard to the realistic approximation of flow-wall interactions, the area of coverage was split into a large number of local boundary condition interfaces. For the outflow, “do nothing” boundary conditions were used.

To assess how well individual areas of the abdominal cavity interact with the incoming flow, also the field values of the local air age (respectively aerosol age) were computed (by operating homogeneous Neumann boundary conditions) for the visceral and the parietal peritoneum. In order to improve the comparability between scenarios with different flow rates, the non-dimensional air age^27,28^ was chosen for data representation. With reference to Eq. (1), the non-dimensional air age τ′ (also known as local air exchange index) is defined as the ratio of the mean air age at the outlet τ_mean,out_ and the local air age τ_local_ within the virtual RCP.

1 $$\tau^{\prime } = \frac{{\tau_{mean,out} }}{{\tau_{local} }}$$

### Ex-vivo/situ aerosol analysis

**Figure 3:**
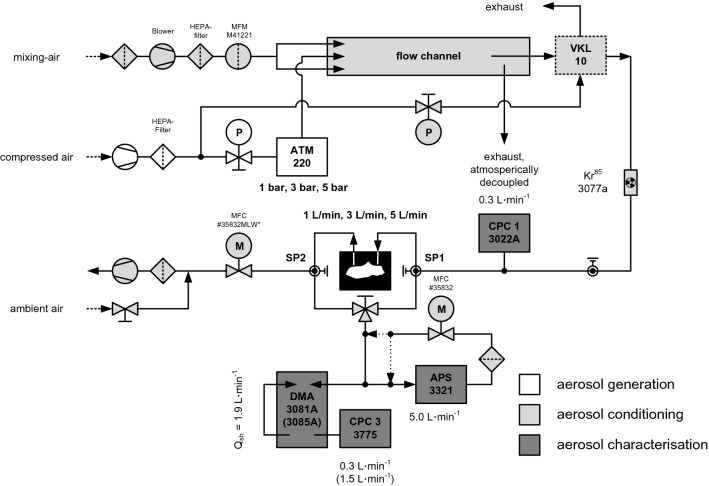
Schematic diagram of the experimental setup for deposition efficiency analyses on the physical rat capnoperitoneum phantom and the alternative phantom.

Ex vivo/situ aerosol deposition analyses within the physical RCP (in supine position) and within the alternative RCP (in upright position) were performed by operating the experimental setup as shown schematically in Fig. 3.

The experimental setup was composed of three functional sections for (i) aerosol generation, (ii) aerosol conditioning (i.e., for modifying concentration and charge condition of the generated aerosol), and (iii) aerosol characterization. An aqueous solution of glucose (Glucosterile® 5%, Fresenius Kabi GmbH, Germany) was used as substance to be aerosolised. Aerosolisation was performed by means of a Collision type^29^ atomizer (ATM 220, Topas GmbH, Dresden, Germany), which was operated with dry (relative humidity < 10%) and particle-free compressed air at system pressures between 1 and 5 bar.

After leaving the atomizer, the primary generated aerosol was pre-diluted within a flow channel that was continuously purged by particle-free air. Required partial flow rates of the aerosol were taken from the flow channel via a commercial aerosol dilution unit (VKL 10, Palas GmbH, Karlsruhe, Germany) that reduces also the concentration of the incoming aerosol by a factor of 10^30^. Prior upstream aerosol analyses, the aerosol was passed through a bipolar radiation charger (85-Kr, model 3077a, TSI Inc., Shoreview, USA) to neutralize the aerosol particles. This was necessary to prevent charging of the inner surface of the non-conductive physical RCP that would lead in the case of unipolar charged aerosols to a decreased particle deposition due to electrostatic repulsion. Beside analyses on the basis of bipolar neutralized aerosols also analyses with unipolar charged aerosols were performed. For these analyses, the effect of intrinsic particle charging during atomisation by spray polarization^31^ was used and no bipolar neutralizer operated.

Aerosol characterisation was performed by different aerosol analytical instruments. The particle number concentration of the upstream aerosol, which was set to approx. 1 × 10^5^ cm^−3^ to avoid changes in the aerosol state by coagulation, was continuously monitored by a condensation particle counter (CPC model 3022A, TSI Inc. Shoreview, USA)^32^, while the charge condition of the aerosol was characterized in secondary analyses by the electrometer of an aerosol length concentration meter (EAD model 3070A, TSI Inc., Shoreview, USA)^33^. To characterise the fractional deposition efficiency (i.e., deposition efficiency over particle size), aerosol samples for analyses were taken alternating from the inlet (sample point SP1, upstream aerosol) and the outlet (sample point SP2, downstream aerosol) of the physical RCP for three times each. Number-weighted particle size distributions and particle number concentrations were analysed over a size range from 0.005 µm up to 20 µm by differential electrical mobility analyses (DEMA) according to ISO 15900:2020^34^ and time of flight spectrometry (TOF, APS model 3321, TSI Inc., Shoreview, USA). For DEMA, two types of electrostatic classifiers (DMA 3081A and DMA 3085A, TSI Inc., Shoreview, USA) and a scientific CPC (CPC model 3775, TSI Inc., Shoreview, USA) were operated.

A vacuum pump in combination with a mass flow controller (MFC, model MLW*, ANALYT-MTC Messtechnik, Müllheim, Germany) was used to simulate different aerosol flow rates (i.e., 1 L/min, 3 L/min, 5 L/min) through the physical RCP as well as through the alternative RCP. Conductive hose-lines and conductive connection elements were used for all aerosol-bearing regions of the setup after dilution. Over all analyses (n = 108), the aerosol showed a relative humidity of 25.39% ± 4.74% and a temperature of 25.67 °C ± 0.57 °C.

## Results and discussion

### In-silico CFD analysis: mean flow velocity fields in coronal and sagittal sectional plane

**Figure 4:**
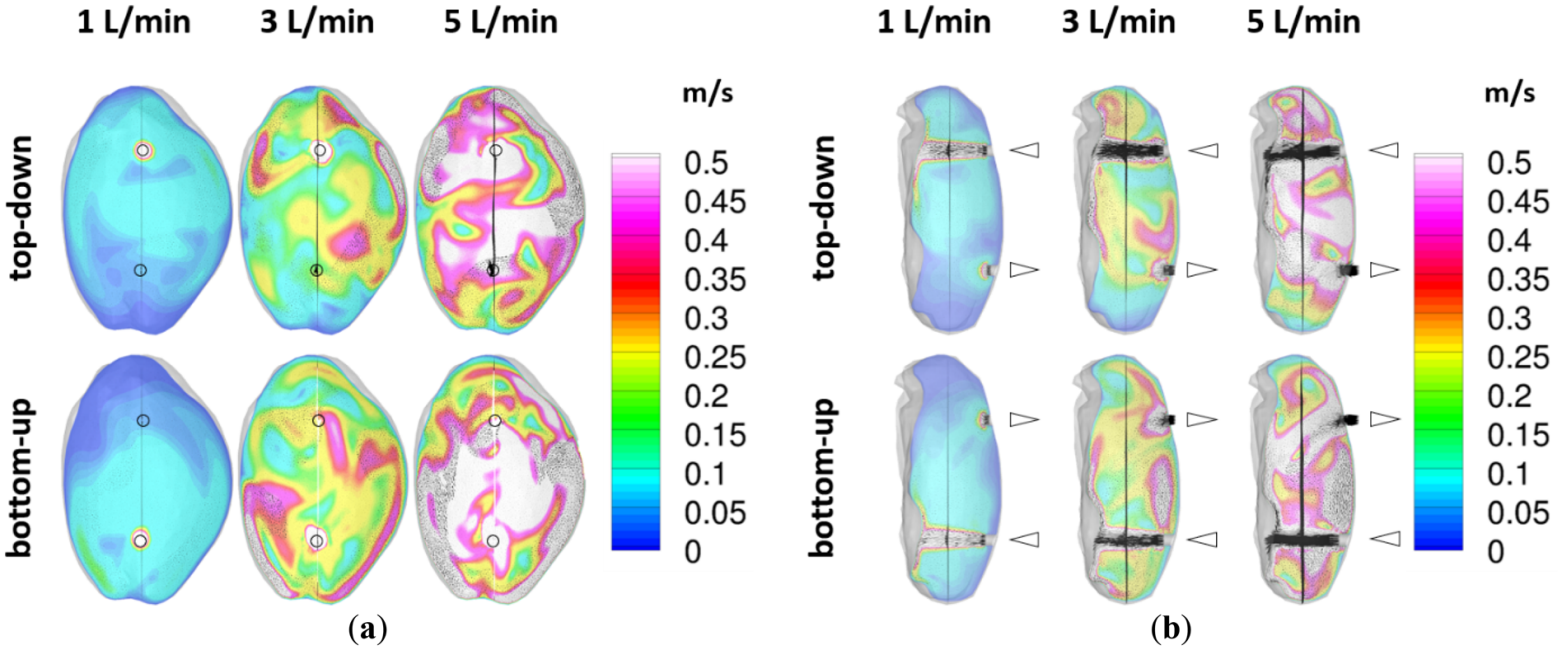
In-silico CFD analysis: mean flow velocity (and flow vectors) in (**a**) coronal sectional plane and (**b**) sagittal sectional plane for top-down and bottom-up purging; black circles indicate position/size of inlet/outlet ports; black triangles indicate flow direction.

Selected results (i.e., for bottom-up and top-down purging at different airflow rates) of the performed in-silico CFD analysis (for nearly steady state air flow conditions reached in any scenario after 30 min simulated time) are shown in Fig. 4.

The comparison of the results for top-down purging and bottom-up purging shows at first significant differences in the mean flow velocity profiles for both the coronal sectional plane (Fig. 4a) and the sagittal sectional plane (Fig. 4b). This holds also true for all analysed flow rates (i.e., 1 L/min, 3 L/min and 5 L/min) and is attributed to the non-symmetric geometric conditions of the visceral peritoneum. At a flow rate of 1 L/min, considerable regions with quasi no perfusion (designated as dead zones of flow) can be observed in both the coronal and the sagittal sectional plane. With increasing flow rate, the extent of the dead zones decreases. For 3 L/min, these dead zones take more space in the case of top-down purging than for bottom-up purging. Quasi no dead zones were observed for 5 L/min.

### In-silico CFD analysis: aerosol age at visceral and parietal peritoneum

**Figure 5:**
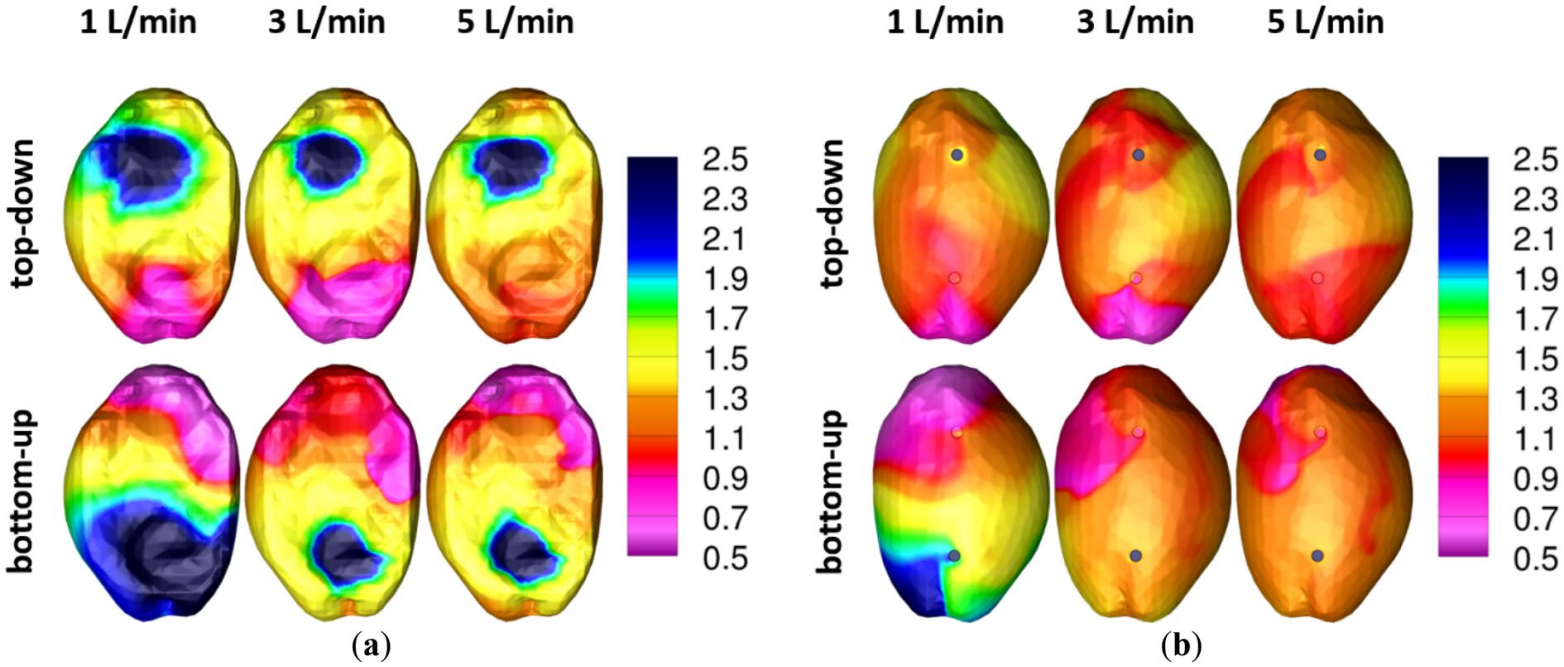
In-silico CFD analysis: (**a**) nondimensional air/aerosol age at visceral peritoneum (left-dorsal-right view); (**b**) nondimensional air/aerosol age at parietal peritoneum (right-ventral-left view).

Figure 5 shows the distribution of the calculated nondimensional air age at the visceral and the parietal peritoneum of the rat capnoperitoneum (after 30 min of simulated time for all scenarios). According to Eq. (1), the non-dimensional air age τ′ becomes < 1 for higher residence times and > 1 for shorter residence times than the mean air age at the outlet.

As expected, Fig. 5a) shows that the regions of lowest residence time (τ’ > 1) prevail on the opposite site of the inlet at the visceral peritoneum. Moreover, it can be observed that these regions become smaller with increasing flow rate. If electrostatic deposition effects do not matter, it is assumed that in these regions rather larger particles will deposit due to inertia than finer particles due to diffusion. Regions with highest residence time (τ′ < 1) are located at the outer areas near the opposite site of the outlet. For these regions a predominantly deposition of finer particles caused by diffusion is assumed, when electrostatic deposition effects are absent.

In contrast to the visceral peritoneum and excepting the scenario of bottom-up purging at 1 L/min, the non-dimensional air age at the parietal peritoneum as shown in Fig. 5b) is even more homogenous distributed and shows quasi no non-dimensional air age hot spots with τ′ > 1.5. Accordingly, it is assumed that the main deposition mechanism acting on the parietal peritoneum is of diffusive nature.

### Ex vivo/situ aerosol analysis: fractional particle separation efficiency

**Figure 6:**
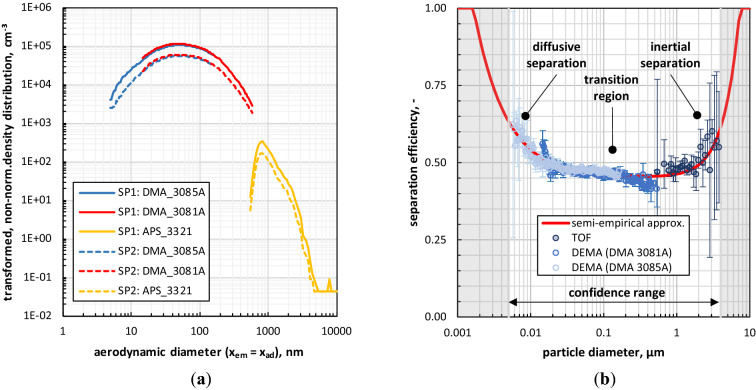
Granulometric results based on ex-vivo/situ aerosol analysis on the physical RCP: (**a**) mean (n = 3) particle size distributions at inlet (SP1) and outlet (SP2) at a volumetric flow rate of 3 l/min; (**b**) separation efficiency data over particle size and corresponding semi-empirical function for a volumetric air flow rate of 3 l/min of the bipolar charged test aerosols; confidence range refers to the size range of adequate measurement data (suggested for data interpolation).

Figure 6a) shows exemplarily the intrinsically measured non-normalized size distributions of the test aerosol at the inlet (SP1, solid lines) and the outlet (SP2, dashed lines) of the physical RCP for a flow rate of 3 L/min of the bipolar neutralized aerosol.

According to Fig. 6a), the size distribution data of TOF supplement well the size distribution data of DEMA. This holds true for both the inlet and the outlet aerosol. By considering the size distribution data of TOF, a peak located at 0.8 µm can be observed. Note that this peak is artificially formed due to the decreasing counting efficiency (i.e., an immanent property of optical instruments) towards the lower detection limit. However, the upstream aerosol showed a monomodal and polydisperse size distribution with a mean count median diameter of x_50,0_ = (56.6 ± 0.89) nm and a mean geometric standard deviation of σ_ln_ = 2.2 ± 0.01. Performed secondary analyses concerning the aerosol charge condition revealed that without bipolar neutralization, spray polarization during atomization lead for the operated 5 wt.-% aqueous glucose solution to a macroscopic positively charged aerosol.

While the granulometric characteristics of the upstream aerosol were largely constant over the experiments, the granulometric downstream aerosol characteristics were affected in a different degree by the occurring particle deposition within the RCP. The degree of particle deposition depends i.a. on (i) the flow conditions trough the RCP, (ii) the charge state of the upstream aerosol, (iii) the electrostatic conditions of the inner RCP surfaces, (iv) the geometric surface conditions and (v) the locations for aerosol supply and exhaust. Accordingly, the differences in the particle size distributions and concentrations of the upstream aerosol and the downstream aerosol (i.e., outlet aerosol) can be used for determining a size-resolved particle deposition efficiency (also called fractional separation efficiency in the area of filter testing). Thus, the deposition efficiency η_dep_(x) was calculated by means of Eq. (2) and the intrinsically measured particle number concentrations at the inlet c_0,SP1_(x) and the outlet c_0,SP2_(x) for each size channel.

2 $$\eta_{dep} \left( x \right) = 1 - \frac{{c_{0, SP2} \left( x \right)}}{{c_{0,SP1} \left( x \right)}}$$

Figure 6b) shows i.a. the calculated mean (n = 3) separation efficiency data for the physical RCP over the particle size on the example of the bipolar neutralized test aerosol at a flow rate of 3 L/min. The representation of these data reflects the typical curve shape of separation efficiency data with three characteristic regions: (i) a plateau-like transition region in the middle with lowest separation efficiencies and a global minimum (also known as most penetrating particle size, MPPS) between (200–600) nm, (ii) a region on the left with an increase of the separation efficiency with decreasing size based on increasing diffusive impact, and (iii) a region on the right, where the separation efficiency increases with increasing size due to increasing inertia.

Furthermore, it can be observed in Fig. 6b) that the characteristic fluctuations of the intrinsically measured size distribution data lead to a strengthen of the spreading in the deposition efficiency data especially for the boundary regions of the size distribution of the test aerosol. To avoid confusion and to improve the clearness of the experimentally determined data, the following semi-empirical approximation was used in this study for linear regression of separation efficiency curves (by combining the method of least squares and the Levenberg–Marquardt algorithm).

3 $$\eta_{dep,RCP,i} \left( x \right) = {\text{min}}\left( {A \cdot x^{ - 1} + B + C \cdot x^{2} ,1} \right)$$

According to Eq. (3), the semi-empirical approximation for the separation efficiency is composed of three terms with three (partly non-dimensionless) modulation parameters (A, B, C). The first term refers to the increasing separation efficiency with decreasing size due to diffusion (~ 1/x), the second term serves to represent the raised transition region and the third term describes the increasing separation efficiency with increasing particle size based on inertia (~ x^2^). A comparison between the separation efficiency data determined directly from measurement data and the approximated regression (coefficient of determination R^2^ = 0.81) is also shown in Fig. 6b).

**Figure 7:**
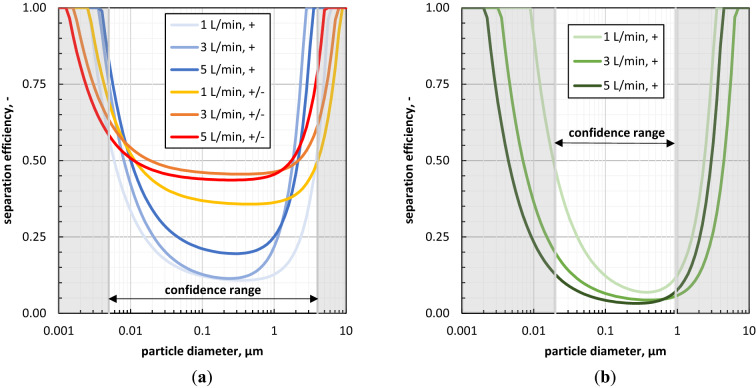
Granulometric results based on ex-vivo aerosol analysis with bipolar neutralized (+/−) and macroscopic positive charged (+) aerosols: impact of the volumetric air flow rate on the separation efficiency within the (**a**) physical RCP and the (**b**) alternative RCP.

The approximated separation efficiency curves for all analysed scenarios of aerosol supply into the RCPs are shown in Fig. 7. Results for the physical RCP are represented in Fig. 7a), while Fig. 7b) provides the results for the alternative RCP. Note that corresponding modulation parameters, coefficients of determination, and most penetrating particle sizes are summarized in Table 2.

**Table 2:** Studied scenarios for drug aerosol supply to the rat capnoperitoneum phantoms (RCP).

Phantom	Flow rate (L/min)	Charge^a^ (–)	A (µm)	B (–)	C (µm^−2^)	R^2b^ (–)	MPPS^c^ (nm)
Physical RCP	1	+/−	1.69E−3	3.53E−1	8.94E−3	0.35	447
	3	+/−	9.03E−4	4.52E−1	1.02E−2	0.81	355
	5	+/−	7.55E−4	4.32E−1	2.24E−2	0.71	251
Physical RCP	1	+	2.37E−3	9.75E−2	2.73E−2	0.40	355
	3	+	3.28E−3	9.31E−2	1.21E−1	0.45	251
	5	+	3.23E−3	1.78E−1	6.93E−2	0.56	282
Alternative RCP	1	+	8.72E−3	3.36E−2	8.20E−2	0.91	398
	3	+	3.32E−3	3.11E−2	2.36E−2	0.80	398
	5	+	2.12E−3	2.04E−2	5.39E−2	0.65	282

^a^i.e., aerosol charge condition; + / − … bipolar neutralized aerosol, + … macroscopic positively charged aerosol.

^b^R^2^ = coefficient of determination.

^c^MPPS = most penetrating particle size.

Prior discussing Fig. 7, it should be noted that the most realistic results of this study are the ones for the bipolar neutralized aerosol and the physical RCP. This is due to the fact that in this scenario the used non-conductive material for the physical RCP does not significantly affects the deposition efficiency.

Considering at first the data for the bipolar neutralised aerosol and the physical RCP as shown in Fig. 7a). It can be observed that with increasing aerosol flow rate (i) the region of inertial separation shifts towards finer particles, (ii) the region of diffusive separation shifts towards finer particles, and (iii) that there is no linear relationship for the location of the plateau. The latter observation is attributed to the contemporaneous decrease of the residence time with increasing aerosol flow rate.

Next to the separation efficiency curves for the bipolar neutralized aerosol, Fig. 7a) shows also the separation efficiency curves on the basis of the macroscopic positively charged aerosol. The direct comparison reveals that the separation efficiency within the physical RCP is significantly lower for the positively charged aerosol than for the bipolar neutralized aerosol. That can be explained by the used non-conductive material of the physical RCP. Thus, deposited particles on the inner surface of the physical RCP form over time a repulsive, positively charged layer that counteracts further particle deposition. Of course, this is an artificial-caused effect of the physical RCP that would not be occur within living or post mortem rats. But these data show impressive the impact of the aerosol charge condition on particle deposition. Moreover, it is assumed that within living or post mortem rats the deposition efficiency is significantly higher for a macroscopic unipolar charged aerosol (i.e., either positively or negatively charged aerosol) than for a bipolar neutralised aerosol. That is attributed to the formation of attractive electrostatic forces at the natural aqueous liquid film on the peritoneum due to the image charge effect.

Determined separation efficiency curves for the alternative RCP (i.e., a simple laboratory glass bottle as described in subchapter 3.2) on the basis of the macroscopic positively charged aerosol are provided in Fig. 7b). The direct comparison of these data with the data of the macroscopic positively charged aerosol for the physical RCP of Fig. 7a) reveal that the deposition results depend strongly on the used phantom. In this example, the alternative RCP shows a significant lower deposition efficiency than determined for the physical RCP. Moreover, the alternative RCP shows an inverse impact with regard to the aerosol flow rate. In the case of the physical RCP with macroscopic positively charged aerosol, the separation efficiency increases with increasing flow rate, while in the case of the alternative RCP a decrease of the separation efficiency can be observed for increasing aerosol flow rate.

### Estimation of particle deposition based on given size distribution data

Fractional separation efficiency η_dep_ curves as determined in this study for the rat capnoperitoneum are independent from the type of quantity r (i.e., specific weighting like by number or mass) and can be used to calculate the total particle deposition in analogy to existing computation models for the respiration tract of animals or humans (e.g. ICRP 66 model^35^ or Multiple Path Dosimetry Model^36^). Therefore, the empirical determined separation efficiency curves η_dep,RCP,i_ have to be multiplied according to Eq. 4 with the fractional quantities of a known particle size distribution q_r_(x), regardless of intrinsically measured data or approximated size distribution data.

4 $$\eta_{dep,tot,r} = \mathop \smallint \limits_{{x_{min} }}^{{x_{max} }} \eta_{dep,RCP,i} \left( x \right) \cdot q_{r} \left( x \right)dx$$

In most cases, particle size distributions of aerosols in the sub-micrometre size range have a right skewed character and can be well approximated by a lognormal distributed density function. According to Eq. (5), the lognormal size distribution density can be described solely by two characteristic parameters, i.e., the median particle size x_50,r_ and the geometric standard deviation σ_ln_.

5 $$q_{r,LNVT} \left( x \right) = \frac{1}{{\sigma_{ln} \cdot \left( {2 \cdot \pi } \right)^{0,5} }} \cdot \frac{1}{x} \cdot {\text{exp}}\left[ { - \frac{1}{2} \cdot \left( {\frac{{{\text{ln}}\left( {x/x_{50,r} } \right)}}{{\sigma_{ln} }}} \right)^{2} } \right]$$

Thus, aerosol size distributions are specified typically in the literature only by the median dimeter, which is equal to the geometric mean diameter for lognormal size distributions, and the geometric standard deviation.

**Figure 8:**
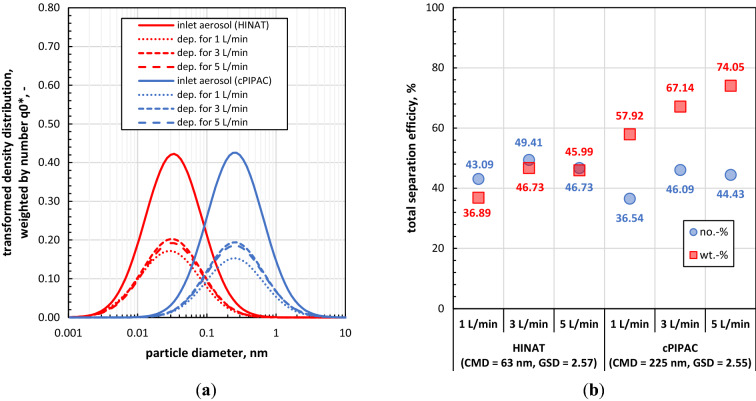
Estimation of particle deposition for bipolar neutralized (+/−) aerosols within the physical RCP on the basis of given size distribution data: (**a**) calculated lognormal size distribution data for two different aerosols with corresponding fractional amounts of deposited particles and (**b**) overall separation efficiency for both aerosols.

In the following calculation example, published number-weighted particle size distribution data^8^ of two different aerosols were used, i.e., for the aerosol of the hyperthermic intracavitary nano-aerosol therapy (HINAT, x_50,0,HINAT_ = 63.4 nm; σ_ln,HINAT_ = 2.57) and for the fine fraction of the aerosol of conventional PIPAC (x_50,0,cPIPAC_ = 254 nm, σ_ln,cPIPAC_ = 2.55). Figure 8a) shows at first the size distribution data of the mentioned aerosols (continuous lines) as calculated by means of Eq. (5). Next to them, Fig. 8a) shows also the fractional amount of the deposited particles within the physical RCP (dotted and dashed lines) for different flow rates, which result from multiplying the size distribution data of Eqs. (3) and (5) for the scenario of the bipolar neutralised aerosol (see Table 2).

Equation 4 in combination with the given size distribution data leads to number-weighted total separation efficiencies as shown in Fig. 8b). To calculate also mass-weighted total separation efficiency data, the number-weighted size distributions have to be converted into volume/mass-weighted ones according to ISO 9276-5:2005^37^ prior using Eq. (4).

## Summary and conclusions

With regard to anticancer research on peritoneal carcinomatosis, rodent cancer models^13–15^ were designed recently to study beside the efficacy of pressurized intraperitoneal chemotherapy (PIPAC)^1^ also the suitability of different drugs and pharmacokinetic effects. In two studies^6,13^, the conventional PIPAC technology for humans was used, while in Rezniczek et al.^15^ a more species-appropriate procedure for drug aerosolization and supply was designed. But mindless of conventional PIPAC or alternative procedures, there are a lot of knowledge gaps concerning drug aerosol supply, drug aerosol propagation and particle deposition. This holds true for the capnoperitonea of both humans and animals.

In order to support anticancer research on peritoneal carcinomatosis, a static rat capnoperitoneum phantom (RCP) was developed. For this purpose, highly resolved CT-images of the capnoperitoneum (at 8 mmHg capnoperitoneal pressure) of a 275 g RNU rat were used to deduce at first a virtual RCP with a capnoperitoneal volume of 146 mL. By means of 3D printing, also a detailed physical RCP made of glycol modified polyethylene terephthalate was created. In comparison to the virtual RCP, the final physical RCP showed a slight lower capnoperitoneal volume of 131 mL that was caused by different technical circumstances. To qualify both the virtual RCP and the physical RCP, comprehensive analyses were performed for a specific PIPAC procedure^8^, where the capnoperitoneum was continuously purged by the drug aerosol.

At first, in-silico analyses by computational fluid dynamic modelling were performed to characterise the impact of different air/aerosol flow rates and the purging direction on the spatial flow velocity distribution within capnoperitoneum and to identify fluidic hot spots on the visceral and the parietal peritoneum. Results show that the non-symmetric geometric conditions of the visceral peritoneum cause a very inhomogeneous spatial flow velocity distribution within the capnoperitoneum and thus also varying local hot spots for residence time on both the visceral and the parietal peritoneum. Moreover, results differ significantly in dependence of the purge direction. Accordingly, the local position of drug aerosol supply should also have a strong impact on the spatial drug distribution.

Ex-vivo/situ aerosol analyses for characterizing size-resolved particle separation efficiencies (over a size range from a few nanometres up to a few micrometres) could be successfully performed under isothermal conditions (i.e., no temperature difference between aerosol and phantom) using the developed physical RCP. The impact of different flow rates and the aerosol charge condition on the fractional particle deposition was analysed. In addition, a simple semi-empirical approximation function for the fractional particle separation efficiency was deduced for the purpose for future data transfer. However, results show a significant impact of the flow rate and the aerosol charge condition on the fractional particle separation efficiency. Moreover, additional analyses by means of a simple alternative RCP (i.e., a laboratory glass bottle with a volume of 135 mL) revealed that geometric-similar phantoms like the developed virtual and physical RCP are of fundamental importance to characterize fractional particle separation efficiencies.

Of course, the developed RCPs show also some limitation. First, both the virtual and the physical RCP are of static nature, i.e., different capnoperitoneal volumes as well as typical movements of the visceral and the parietal peritoneum cannot be mimicked yet. Second, the physical RCP was made of non-conductive material. To mimic more realistic conditions within the RCP, an electrically and thermal conductive material has to be used and/or a wetting of the inner surface of the RCP is necessary. The latter requirement can be performed i.a. similar to Buggisch et al.^38^ by coating the surface with nitrocellulose membranes soaked with 0.9 wt.-% aqueous sodium chloride solution or by coating the surface with agar. Third, only one transport equation was used in this study to characterize fluid dynamic phenomena within the virtual RCP. Accordingly, presented data are representative for steady state conditions either for air supply or for aerosol supply. Computing of aerosol propagation on the basis of different transport equations for air/carbon dioxide and aerosol as performed for example by Göhler et al.^28,39^ can significantly improve the model.

Finally, the conducted procedures for the development of the rat capnoperitoneum phantom as well as the in-silico and ex-vivo/situ analytical methods can be transferred in future work also to the capnoperitonea of other animals (like mice or pigs) or directly to the ones of humans.

## Supplementary Information

Supplementary Video 1.

## Data Availability

Requests on analytical data and on the CAD files of the RCP should be addressed to D.G.

## Abbreviations

APS: Aerodynamic particle sizer
CAD: Computer aided design
CFD: Computational fluid dynamic
CPC: Condensation particle counting/counter
CT: Computed tomography
DEMA: Differential electrical mobility analysis
EAD: Electrical aerosol detector
HINAT: Hyperthermic intracavitary nano-aerosol therapy
MFC: Mass flow controller
MFM: Mass flowmeter
MPPS: Most penetrating particle size
PIPAC: Pressurized intraperitoneal aerosol chemotherapy
RCP: Rat capnoperitoneum phantom
SMPS: Scanning mobility particle sizer
STL: Stereolithography or standard triangle/tessellation language
TOF: Time-of-flight spectrometry
